# Supplementary material for: The Roles of Gut Microbiome and Plasma Metabolites in the Associations between ABO Blood Groups and Insulin Homeostasis: The Microbiome and Insulin Longitudinal Evaluation Study (MILES)
Source: Metabolites. 2022 Aug 25;12(9):787. doi: 10.3390/metabo12090787 (PMC9505353; doi:10.3390/metabo12090787)
Supplement: Supplementary file 1 [file metabolites-12-00787-s001.zip › metabolites-1874217-supplementary.pdf]

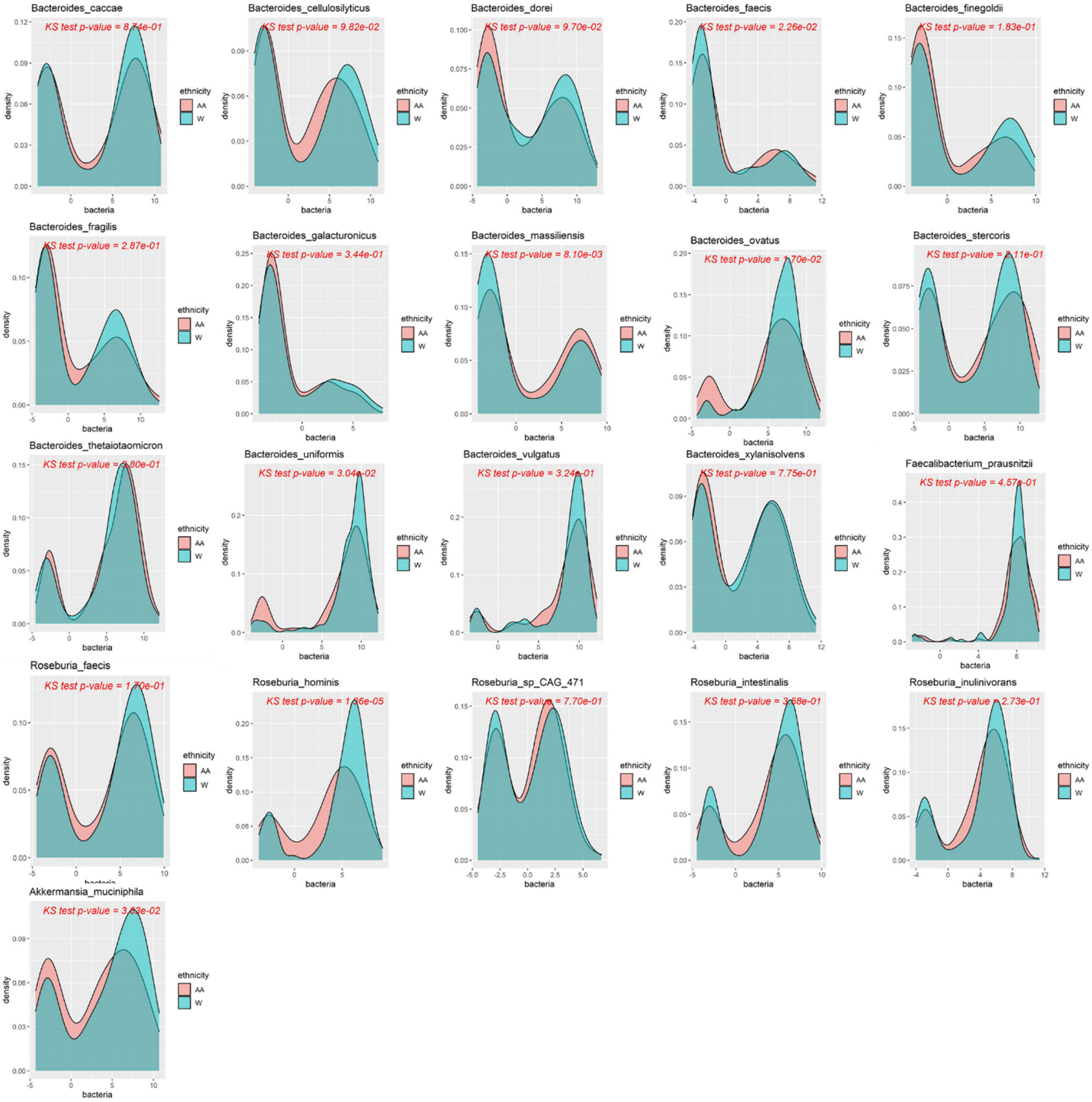


**Figure S1.** The relative abundance distributions of 21 selected bacterial species between Whites and African American subpopulations.

**Table S1.** Correspondence of ABO haplotype determined by single nucleotide polymorphisms (SNPs).

| SNP RS ID | TAG ABO HAPLOTYPES |
|---|---|
| **RS8176719-C INSERTION** | O1 |
| **RS41302905-T** | O2 |
| **RS1053878-A** | A2 |
| **RS8176743-T** | B |
| **RS579459-C** | A1 |
| **RS2519093-T** | A1 |

**Table S2.** HD4 chromatographic method summary.

| | | | | |
|---|---|---|---|---|
| **NEG** | | | | |
| Time (min) | Flow (mL/min) | %A | %B | A=6.5mM Ammonium Bicarbonate Aqueous pH ~8 |
| Initial | 0.35 | 99.5 | 0.5 | B= 6.5mM Ammonium Bicarbonate in 95%Methanol 5% Water |
| 4 | 0.35 | 30 | 70 | |
| 4.5 | 0.35 | 1 | 99 | Injection solvent=6.5mM Ammonium Bicarbonate in water |
| 5.5 | 0.35 | 1 | 99 | |
| 5.7 | 0.35 | 99.5 | 0.5 | |
| 6.8 | 0.35 | 99.5 | 0.5 | |
| **POSear** | | | | |
| Time (min) | Flow (mL/min) | %A | %B | A=Water 0.1%Formic acid 0.05% Perflouropentanoic acid (PFPA) |
| Initial | 0.35 | 95 | 5 | B=Methanol 0.1%FA 0.05% Perflouropentanoic acid (PFPA) |
| 3.35 | 0.35 | 20 | 80 | |
| 3.36 | 0.35 | 95 | 5 | Injection Solvent= Water 0.1%Formic acid 0.05% Perflouropentanoic acid (PFPA) |
| 3.4 | 0.35 | 95 | 5 | |
| **POSLate** | | | | |
| Time (min) | Flow (mL/min) | %A | %B | A=Water 0.1%Formic acid 0.05% Perflouropentanoic acid (PFPA) |
| Initial | 0.6 | 60 | 40 | B= 50%Acetonitrile 50% Methanol 0.1%Formic acid 0.05% Perflouropentanoic acid (PFPA) |
| 1 | 0.6 | 0.5 | 99.5 | |
| 3.4 | 0.6 | 0.5 | 99.5 | Injection Sovent = 90%Isopropanol 10% water with 0.1%Formic acid 0.05% Perflouropentanoic acid (PFPA) |
| 3.41 | 0.6 | 60 | 40 | |
| **Pol** | | | | |
| Time (min) | Flow (mL/min) | %A | %B | A=10mM Ammonium Formate in 15%water 5%Methanol 80%Acetonitrile ph ~10.16 |
| Initial | 0.5 | 95 | 5 | B=10mM Ammonium Formate in 50%water 50%Acetonitrile ph ~10.6 |
| 3.5 | 0.5 | 50 | 50 | |
| 5.5 | 0.5 | 5 | 95 | Injection solvent= 10mM Ammonium Formate in 15%water 5%Methanol 80%Acetonitrile ph ~10.16 |
| 6.5 | 0.5 | 5 | 95 | |
| 6.7 | 0.5 | 95 | 5 | |

**Table S3.** HD4 Standards.

| | **Instrument Performance Standards** | **Process Assessment Standards** |
|---|---|---|
| **POSear (Method 1)** | d7-Glucose | Flourophenylglycine |
| | d5-Glutamine | Chlorophenylalanine |
| | d2-Threonine | |
| | d5-Hippuric Acid | |
| | d3-Methionine | |
| | d3-Leucine | |
| | Bromophenylalanine | |
| | | |
| | | |
| **POSLate (Method 2)** | Bromophenylalanine | d6-Cholesterol |
| | d5-Androstene | Chlorophenylalanine |
| | d9-Progesterone | |
| | d4-Dioctylphthalte | |
| | | |
| | | |
| **Neg (Method 3)** | d7-Glucose | Tridecanoic Acid |
| | d3-Methionine | Chlorophenylalanine |
| | d3-Leucine | |
| | d8-Phenylalanine | |
| | d5-Tryptophan | |
| | Bromophenylalanine | |
| | d15-Octanoic Acid | |
| | d19-Decanoic Acid | |
| | d27-Tetradenanoic Acid | |
| | d35-Octadecanoic Acid | |
| | d2-Eicosanoic Acid | |
| | | |
| **Pol (Method 4)** | d35-Octadecanoic Acid | Chlorophenylalanine |
| | d5-Indole Acetate | Flourophenylglycine |
| | Bromophenylalanine | d8-Valine |
| | d5-Tryptophan | |
| | d4-Tyrosine | |
| | d3-Serine | |
| | d3-Aspartic Acid | |
| | d7-Ornithine | |
| | d4-Lysine | |

**Table S4.** Mass accuracy summary.

| Name | Average error (ppm) |
|---|---|
| Isoleucine | 0.96 |
| Leucine | 0.79 |
| Valine | 0.26 |
| Lactate | 6.21 |
| Glucose | 0.69 |
| 1,5-AG | 0.35 |
| 2-Hydroxybutyrate/iso | 2.32 |
| N-lactoyl Phenylalanine | 0.41 |
| N-lactoyl Tyrosine | 0.70 |
| N-lactoyl Valine | 0.53 |
| N-lactoyl Leucine | 0.86 |
| N-lactoyl Isoleucine | 0.63 |
| Metabolonic Lactone Sulfate | 0.37 |


| **Name** | **Average error (ppm)** |
|---|---|
| Isoleucine | 0.96 |
| Leucine | 0.79 |
| Valine | 0.26 |
| Lactate | 6.21 |
| Glucose | 0.69 |
| 1,5-AG | 0.35 |
| 2-Hydroxybutyrate/iso | 2.32 |
| N-lactoyl Phenylalanine | 0.41 |
| N-lactoyl Tyrosine | 0.70 |
| N-lactoyl Valine | 0.53 |
| N-lactoyl Leucine | 0.86 |
| N-lactoyl Isoleucine | 0.63 |
| Metabolonic Lactone Sulfate | 0.37 |
